# Diabetes Care and Education: A Look Backward and Forward

**DOI:** 10.17925/EE.2025.21.1.5

**Published:** 2025-03-12

**Authors:** Linda M Siminerio, Youjia Wang, Denise Charron-Prochownik

**Affiliations:** 1. University of Pittsburgh Medical Center, Pittsburgh, PA, USA; 2. Department of Medicine and Department of Nursing, Health and Community Systems, University of Pittsburgh, Pittsburgh, PA, USA; 3. Department of Health Promotion and Development, School of Nursing, University of Pittsburgh, Pittsburgh, PA, USA

**Keywords:** Behavioural, diabetes, education, evidence-based, health systems, psychosocial

## Abstract

The importance of self-management and education is now generally known and accepted in the diabetes community. Despite this, the number of people with diabetes who receive diabetes education and psychosocial services continues to be disappointing. While clinical advances are being adopted, referrals to diabetes education remain low, and resources for behavioural support are scarce. This calls for a need to inform and remind care providers and healthcare decision-makers of the efforts of all those who built the foundation for comprehensive diabetes care, which continues to inform practice and serve as a backdrop for research to address today’s challenges.

Diabetes is a chronic disease associated with both acute and chronic complications. Many advances have been introduced throughout history to address these problems. While each clinical breakthrough was welcomed with relief and the expectation that a solution had been discovered, it was followed by the acknowledgement that continued exploration was needed. The scientific trials and tribulations of the past did offer a stage for today’s exciting innovations. Early studies made it clear that positive clinical outcomes also depend on the delivery and access to quality care, education and self-management, opening new areas of study.

## The advent of self-blood glucose monitoring

During the darkest period of diabetes care, before the life-saving discovery of insulin in 1921, treatment consisted of near-starvation diets, and patients suffered from multiple morbidities and untimely mortality. The discovery of insulin in 1921 was followed by the continued development of improved insulins and delivery methods, along with the introduction of sulfonylureas and a series of innovative mechanistic approaches in the treatment of type 2 diabetes.^1^ Self-blood glucose monitoring (SMBG) was later heralded as the solution to the problems associated with diabetes in the late 1970s.^1^ Despite the hype at the 1986 American Diabetes Association SBGM Consensus Conference, however, reports on the benefits of SMBG, were disappointing.^2^ The Consensus Committee advised the advancement of the technology, assurance of accuracy, limiting chances for user error and the establishment of algorithms for adjusting insulin to titrate diet, exercise and/or medication accordingly.^3^ The Committee emphasized that results are meaningless if people do not learn how to react to the values.^3^ The healthcare community was strongly advised to provide patient education that included self-regulation of their glycaemia in using SMBG and insulin dose self-adjustment, and that more research was needed.^2,3^

Industry responded to the Consensus Committee by improving blood glucose testing technology with more sophisticated and user-friendly devices. Disappointingly, however, at the same time, a published study suggested that diabetes education was ineffective.^4^ A randomized controlled trial reported that education may not be an effective therapeutic intervention for most adults with insulin-treated diabetes.^4^ In a large group of patients who received education versus those in usual care, knowledge scores improved; however, there was no improvement in clinical measures of haemoglobin A1c (HbA1c) and blood pressure.^4^ Although this was discouraging, it conveyed an important message to the diabetes education and research community that continued research efforts were needed in building the evidence. The response to the study findings was summarized: ‘The effects of educational programmes are of limited value if they do not lead to permanent changes in attitudes and motivation, critical factors affecting long-term diabetic control’.^5^ Measures of successful education should not be limited to improvements in knowledge alone; HbA1c levels, intermediate outcomes and long-term outcomes, such as changes in health beliefs and attitudes, adherence, behaviour change, self-efficacy and quality of life (QOL), need to serve as endpoints that deserve consideration demonstrated in a series of studies.^6–12^

## Dawning of the psychosocial implications of diabetes and team care

Behavioural scientists and educators began efforts for the study of behaviour, psychosocial themes and health systems research and advanced the field of study.^13–18^ Findings from their research began to change attitudes towards patients and practice. The international diabetes community took interest, and the seminal Diabetes Attitudes, Wishes and Needs (DAWN) study was conceived of and designed by behavioural leaders in the field: Richard Rubin, Mark Peyrot and Sven Skovlund. The DAWN study was launched to explore the attitudes, wishes and needs of patients with type 1 and 2 diabetes, physicians and nurses in 11 countries.^19–21^

The results from DAWN provided insights into both patient and practice behaviours. Overall study findings showed a global gap between the psychosocial needs of people with diabetes and the support provided by their care systems – reported issues included poor selfmanagement, coping, QOL, glycaemia and severe complications leading to disability and depression.^22–24^ Follow-up DAWN studies were conducted. A study was then conducted that examined individual- and country-l evel patterns in both patient and provider perceptions of diabetes care. These were examined in 5,104 randomly selected adults with type 1 or type 2 diabetes and in 3,827 randomly selected diabetes care providers, including primary-care physicians (n=2,070), diabetes specialist physicians (n=635) and nurses (n=1,122).^25^ The relationships between outcomes and both country and respondent characteristics were analysed using multivariate analysis, along with the interaction between these two factors. Providers rated both chronic-care systems and the remuneration for chronic care as mediocre, and indicated that several specialist disciplines were not readily available to them. Patients reported that, while there were high levels ease of access to care, it was not without financial barriers; patients with fewer socio-economic resources and more complications from their diabetes had lower access (and/or higher barriers) to care and a lower quality of patient–provider collaboration. Patient-reported outcomes and country-respondent demographic, disease characteristics and healthcare features were all associated.^26^ Better patient–provider collaboration was associated with more favourable ratings on all outcomes, and better access to providers and team care availability were associated with positive outcomes. Physicians who participated in the study stated that better communication should be available to patients, along with increased access to psychologists and qualified nurse educators/specialist diabetes nurses.^25,26^

The important role of nurses was reaffirmed in DAWN research through studies conducted by Martha Funnell and Linda Siminerio.^14,20,26^ Nurses were reported to provide feelings of hope, discuss adherence, act as an intermediary between clinicians and team members and address the critical psychosocial needs of the patient. When patients reported having access to a nurse, they had better outcomes. Unfortunately, however, less than half of the patients surveyed had access to nurse services, and in many countries, nurses had limited scopes of practice.^20,26^

Team care and advancing the roles of its members gained more attention. In a meta-analysis of diabetes care outcomes, the best predictor of improved glycaemia was access to a team and case management.^27^ Nurses had been reported to show a willingness to take on more responsibility for treatment regimens, with additional training.^26^ Nurses’ roles, along with dieticians and pharmacists, continued to expand as research demonstrated their ability and effectiveness in providing guidance and support for medication management.^28–31^ This has enormous potential in providing much-needed access to team-based speciality care and support for primary care in underserved community settings.^32^

## Diabetes self-management education

Studies on the effectiveness of diabetes education were also encouraging and gaining momentum, especially those focused on Diabetes Self-management Education (DSME).^18,33–36^ A comprehensive review revealed that DSME was associated with a dramatic decline in HbA1c levels by as much as 0.76%.^31^ The effectiveness was shown to be directly correlated with the amount of time spent with the diabetes educator. However, like any therapy, the benefits waned over time without continued dosing. Additional studies confirmed the need for ongoing support, now referred to as DSMES (Diabetes Self-Management Education Support). DSMES resources, such as D-SMART (Diabetes Self-management Assessment Report Tool), were developed to assist the educator in this process.^37,38^ Recognizing the importance of DSMES delivered by diabetes educators and their demonstrated ability for therapeutic management support, the formerly termed ‘diabetes educator’ is now referred to as the Diabetes Care and Education Specialist, referencing their role in therapeutic management, along with education.

To ensure that effective methods were available to guide the growing research, psychosocial and care delivery frameworks were created providing road-maps for future research. Russell Glasgow developed the widely used Reach, Effectiveness, Adoption, Implementation, Maintenance (RE-AIM) framework as a template for the development of care delivery programme implementation and evaluation.^39^ Rubin and Peyrot, recognized as pioneers in the psychosocial and behavioural fields, developed and applied qualitative methods for studying these areas.^23,25,40–42^ Behavioural theory-based interventions were adapted or created for youth or culturally adapted for ethnic communities. Margaret Grey’s coping skills training intervention for youth had long-l asting effects on metabolic control and QOL.^43^ Barbara Anderson found that family sharing of diabetes responsibilities for adolescents improved diabetes self-management and metabolic control.^44,45^ Funnell and Robert Anderson developed a programme of research based on empowering individuals with diabetes with better self-management.^46^ In one study in African American adults with type 2 diabetes, an empowerment-based diabetes self-management support intervention was promising for improving and/or maintaining diabetes-related health, particularly HbA1c levels.^47^

Culturally tailored behavioural-diabetes programmes and age-appropriate adaptations to address the needs of specific populations became imperative. For example, Stopping GDM is a dyadic educational-behavioural gestational diabetes prevention programme culturally specific for American Indian and Alaska Native adolescents and their mothers.^48^ Studies focused on transitioning young adults have also been highly effective.^49^

## Access to comprehensive quality care

Specific attention to participant recruitment efforts was made to assure that the population engaged in the study represented people who could benefit from the research findings.^50–54^ Healthcare delivery processes were examined using the Patient-Centred Medical Home and Chronic Care models.^55–58^ Studies conducted by behavioural researchers like David Marrero, Alan Delamater, William Polonsky and Larry Fisher reaffirmed that attention to psychosocial needs is critically important, reorganization of care delivery systems providing team-based care and attending to the specific needs of the population served is essential, DSMES is cost-effective, telemedicine provides access to enhanced care and education, databases and repositories offer a unique resource for data-driven decisions and population health through risk stratification and personal devices like continuous glucose monitor (CGM) and mobile applications offer great opportunities for enhanced self-management.^21,51–54,59–74^

## Applied lessons into practice

Technology is quickly and constantly evolving and over recent years has become an integral part of diabetes care.^75^ While both people with diabetes and clinicians are harnessing a variety of technologies, access issues continue for some populations and efforts are needed in making sure that there are widespread opportunities for access.^76^ CGMs, insulin pumps, automated insulin delivery systems, data-sharing platforms, telehealth, remote monitoring and smartphone mobile applications are being used and shown to improve clinical outcomes and QOL.^74,77–82^ Artificial intelligence technologies are already being integrated into diabetes education interventions for dietary and exercise and insulin injection guidance, monitoring of complications and self-management.^83^

Caring for persons with diabetes who use technology is best accomplished in partnership with other members of the care team and support staff. Although the use of technology is associated with improved outcomes, this effect is enhanced when the user is knowledgeable and engaged; simply wearing a device or just downloading an app may not translate into health benefits.^75,84^

Although the overall wellbeing of those with diabetes has improved considerably over the past several decades, the health of racial and ethnic minorities and other populations with diabetes continues to lag.^85^ For decades, there have been declarations identifying the correction of health disparities as a national priority in the USA. While progress has been slow in meeting the comprehensive needs of all those living with diabetes, new approaches have been tested to improve diabetes self-management and provide support for people with diabetes in vulnerable, underserved populations and settings. Broad-based approaches have been used to identify the scope of the problems, technological advances applied in underserved areas, while patient psychosocial and behavioural challenges have been examined in specific ethnic populations.^53,62,86,87^ Interventions including telehealth, mobile app technology, advanced practice provider-l ed clinics and support from community health workers are finally gaining attention.^9,28,35,71,74,88,89^

## In closing

Over 20 years ago, the National Diabetes Education Programme (NDEP) was launched. It was a programme supported by the NIH and the CDC to translate findings from diabetes research into public and clinical health practice, at a time when the burden of care was on the providing physician, the approach to patient care was directive and patients were expected to do as they were told.^90^ There was no recognition of the psychosocial needs of the person living with diabetes, such as depression and distress. It is unlikely that the founders of NDEP could have predicted the rising rates of diabetes, the science and technology available today and that healthcare disparities would continue. Findings from behavioural and translational science informed the diabetes community that living with a chronic disease takes an emotional toll; the patient being the centre of care and the use of team approaches are the best predictor of success. While progress has been made, challenges remain that demand the attention of and continued efforts from the diabetes clinical and research communities.
